# Use of incretin receptor agonists in patients submitted to metabolic bariatric surgery – a systematic review and meta-analysis

**DOI:** 10.3389/fendo.2026.1870008

**Published:** 2026-08-26

**Authors:** Mariana Santos-Pereira, João F. Frutuoso, Sofia S. Pereira, Marta Guimarães, Mariana P. Monteiro

**Affiliations:** 1 Unit for Multidisciplinary Research in Biomedicine, School of Medicine and Biomedical Sciences,University of Porto, Porto, Portugal; 2 Laboratory for Integrative and Translocation Research in Population Health (ITR), University of Porto, Porto, Portugal; 3 Department of General Surgery, Hospital São Sebastião, Unidade Local de Saúde de Entre o Douro e Vouga, Santa Maria da Feira, Portugal

**Keywords:** incretin receptor agonists, metabolic bariatric surgery, obesity, pharmacotherapy, weight loss

## Abstract

**Introduction:**

Metabolic bariatric surgery is a highly effective weight-loss intervention. Nevertheless, a non-negligible proportion of patients experience a suboptimal clinical response, which may present as insufficient weight loss or weight regain after an initial loss. In these cases, weight management pharmacotherapy can be considered. We aimed to conduct a systematic review and meta-analysis of the best available data on the use of incretin receptor agonists, liraglutide, semaglutide, and tirzepatide, in patients previously submitted to metabolic bariatric surgery.

**Methods:**

We searched PubMed, Scopus, and Web of Science, for original studies that evaluated weight loss and adverse events in adults submitted to metabolic bariatric surgery one year or more before receiving treatment with these drugs.

**Results:**

Twenty-seven papers (n=27) were included in this systematic review, of which nineteen (n=19) were used in the meta-analysis. After treatment with incretin receptor agonists, patients who underwent metabolic bariatric surgery, experienced significant weight loss. Meta-analysis showed, at 12 months, a total weight loss of 9.22% with liraglutide and of 9.02% with semaglutide. At 6 months, a mean difference in total weight loss of 4.23% was presented in favor of tirzepatide as compared to semaglutide. No serious adverse events were observed and most reported side effects were non-serious, consisting primarily of mild gastrointestinal symptoms.

**Discussion:**

This data suggests that incretin receptor agonist treatment after metabolic bariatric surgery demonstrated the ability to achieve significant weight loss with a good safety profile, particularly in patients with a suboptimal clinical response, and thus may serve as an alternative rescue therapy to revisional surgery. However, given the substantial heterogeneity across studies, these findings should be interpreted with caution.

**Systematic Review Registration:**

https://www.crd.york.ac.uk/prospero/display_record.php?ID=CRD42022371697, identifier CRD42022371697.

## 1 Introduction

Metabolic bariatric surgery is an effective intervention to achieve sustained weight loss (WL) (1). Although most patients achieve and maintain adequate WL after metabolic bariatric surgery, 15-35% fail to meet WL targets (2). Additionally, 25-35% can experience significant weight regain (WR) within 2 to 5 years post-surgery (3–5). Nevertheless, it should be highlighted that these estimates present a wide variation across studies due to differences in how “suboptimal clinical response” is defined, whether as primary, i.e. insufficient weight loss (IWL), or secondary, i.e. WR, as well as the variable WL thresholds and the timeframes used to assess outcomes (6, 7). According to the International Federation for the Surgery of Obesity and Metabolic Disorders (IFSO) guidelines, a primary suboptimal clinical response is defined as either a total body weight of less than 20% or inadequate remission of an obesity-associated disorder. A secondary suboptimal clinical response is defined as weight regain exceeding 30% of body weight nadir after surgery or worsening of an obesity-associated disorder that indicated surgery after initial improvement (8). Additionally, it is also important to note that most studies do not present data analysis according to the type of surgery performed, which is a considerable source of variability, given that WL, as well as the probability of suboptimal clinical response, are highly dependent on the bariatric technique performed (9).

Over the past decade, incretin receptor agonists have been introduced as obesity management drugs, including glucagon-like peptide-1 receptor agonists (GLP-1RA) and a dual GLP-1 and gastric inhibitory polypeptide (GIP) receptor (GLP-1R/GIPR) agonist. These drugs have been shown to induce over 5-20% WL and significantly improve obesity-associated disorders (10). GLP-1RAs act by reducing hunger, thus contributing to decreased food intake and ultimately WL (11). GIPR agonism seems to amplify the GLP-1RA’s actions and may contribute to a more physiological and healthier storage of excess lipids (12). Among these, liraglutide, a short-acting GLP-1RA, at a 3.0 mg daily dose, was shown to be effective at achieving a body weight reduction of 5-10% (13). Semaglutide, a long-acting GLP-1RA, at a 2.4 mg weekly dose, was shown to result in a body weight reduction of approximately 15% (14). Tirzepatide, the first-in-class dual GLP-1R/GIPR agonist (15), at a 15 mg weekly dose, was shown to achieve a WL of approximately 21% (16).

In cases of a suboptimal clinical response, treatment with incretin receptor agonists can be considered. The available evidence on the use of these drugs for obesity management in patients previously submitted to metabolic bariatric surgery in comparison to the non-bariatric population is still limited, given that metabolic bariatric surgery was a prespecified exclusion criterion in randomized controlled trials conducted while these drugs were under development. The majority of the available evidence on the use of incretin receptor agonists in patients previously submitted to metabolic bariatric surgery is derived from small-scale academic clinical trials, case-controlled studies, and case series, whose data have been compiled by systematic reviews (17–19). However, given that this is a rapidly evolving field, an updated synthesis of the evidence was warranted, thus, we conducted an updated systematic review and meta-analysis to comprehensively compile the best available and most recent data on the use of incretin receptor agonist drugs in patients with suboptimal clinical response at least one year after metabolic bariatric surgery.

## 2 Materials and methods

### 2.1 Protocol and registration

This protocol was registered at PROSPERO (registration number CRD42022371697) and is accessible at https://www.crd.york.ac.uk/prospero/display_record.php?ID=CRD42022371697.

The literature review protocol was conducted in compliance with the PRISMA guidelines.

### 2.2 Search strategy

Original papers evaluating the use of incretin receptor agonist drugs in patients previously submitted to metabolic bariatric surgery, incorporating the use for IWL or WR, were included in this systematic review. This search was conducted on three different bibliographic databases: PubMed, Scopus, and Web of Science, in June 2025. The search criteria for each database are listed in **Supplementary File 1**.

### 2.3 Selection process and criteria

Pre-established conditions for publications retrieval were: only original studies (controlled and observational), randomized controlled trials, cohort studies, and case series. Only human species and only studies written in English language were included. No date restrictions were applied. The inclusion and exclusion criteria were designed following the PICOS (Patient, Intervention, Comparison, Outcome, Study) format. Only studies conducted in adults who have undergone metabolic bariatric surgery were included. The exclusion criteria included studies conducted in children (under 18 years old), pregnant women, meeting or conference abstracts, unpublished studies, study designs other than controlled and observational, studies not including patients submitted to metabolic bariatric surgery, studies with drug treatment initiated within one year after metabolic bariatric surgery or unknown, studies not providing or evaluating the weight outcomes before or after metabolic bariatric surgery or drug treatment, studies reporting aggregated WL outcomes of different drugs, or studies evaluating pharmacotherapy before metabolic bariatric surgery. The full text version of the article by Medhati et al., 2025 was requested from the corresponding author by email.

### 2.4 Records screening

References were retrieved from the bibliographic databases and imported into an EndNote library. After duplicate removal, article titles and abstracts were screened for eligibility by three independent reviewers (MSP, JFF, SSP). In some cases, full-text examination was required. A fourth member was available to solve any disagreements (MPM). In a second phase, the full text of the records selected as potentially eligible was assessed for study inclusion.

### 2.5 Quality assessment

Quality and risk of bias of each selected paper were determined using the Jahad scale for randomized controlled trials, the Methodological Index for Non-Randomized Studies (MINORS) for cohort studies, and the Joanna Briggs Institute Critical Appraisal Checklist for case series. Only studies scored to be at least moderate quality with the Jahad scale and Joanna Briggs Institute Critical Appraisal Checklist were included. According to the MINORS scale, studies with a score of ≤8 are considered poor quality, 9–14 moderate quality, and 15–16 good quality for non-comparative studies. For comparative studies, the scores were ≤14, 15–22, and 23–24, respectively. After applying the MINORS scale, some studies scored low quality in some parameters because they were retrospective studies in design, included exclusion or details about the exclusion criteria, did not describe or implement blinded assessment (often due to reliance on objective outcomes), or displayed a loss to follow-up higher than 5% or not fully explicit.

### 2.6 Data extraction, synthesis and analysis

A spreadsheet was created to summarize the following data: article, author(s), study design, country, subgroups, number of subjects enrolled, most relevant patient characteristics (age, gender proportion, patients’ complications, weight and body mass index (BMI) before surgery or before therapy), type of metabolic bariatric surgery, time span between metabolic bariatric surgery and drug treatment, criteria used to define a suboptimal clinical response (IWL and/or WR), weight variation after drug treatment, side effects, drug dosage and duration of treatment, comparison with other groups and control, main outcomes. Data retrieval was reviewed by a second author in a cross-over manner (SSP).

The papers included were subdivided into the following groups: (1.1) patients treated with liraglutide, (1.2) patients treated with semaglutide, (1.3) patients treated with tirzepatide, (1.4) studies that compared the outcomes of different drugs.

Data is described in the results section, focusing primarily on the WL outcomes, and secondarily on the adverse effects of drug treatment. Ultimately, this review summarizes the best available evidence on the use of incretin receptor agonists in patients submitted to metabolic bariatric surgery.

### 2.7 Statistical analysis

The retrieved data of WL outcomes after drug treatment for each study were used to calculate the percentage of total weight loss (%TWL) with a 95% confidence interval (CI). In some studies, the WL outcomes were presented as median and interquartile range, and for those, the sample mean and standard deviation were estimated using the estimation method presented by Wan et al. (20). Additionally, baseline weight was defined as body weight at initiation of pharmacological intervention. Therefore, %TWL represented the additional WL achieved during pharmacotherapy and was not calculated relative to preoperative weight or post-bariatric weight nadir. A random-effects meta-analysis model was adopted because of heterogeneity among studies in sample characteristics, type of metabolic bariatric surgery, method of WL assessment, time since surgery, and incretin receptor agonist dosing. Adverse events were analyzed as the proportion of participants experiencing at least one adverse event. Separate random-effects meta-analyses of proportions were conducted for participants experiencing at least one adverse event and were reported separately for each adverse event. Only studies providing an event count and denominator, or a percentage together with the corresponding denominator, were included. Studies reporting combined outcomes, such as nausea and vomiting, were excluded from the corresponding symptom-specific analyses. In studies including multiple pharmacological treatments, only treatment-specific data were used. Missing or unreported adverse-event outcomes were not considered zero events. The results were graphically presented as forest plots with 95% CI. Heterogeneity was assessed using Higgin’s I^2^, and Tau squared (τ^2^). A value I^2^ > 50% implied a substantial heterogeneity between the eligible studies (21). Meta-analysis was conducted in R software version 4.3.0 (R Foundation for Statistical Computing, Vienna, Austria) for Windows.

## 3 Results

### 3.1 Literature search

**Figure 1:**
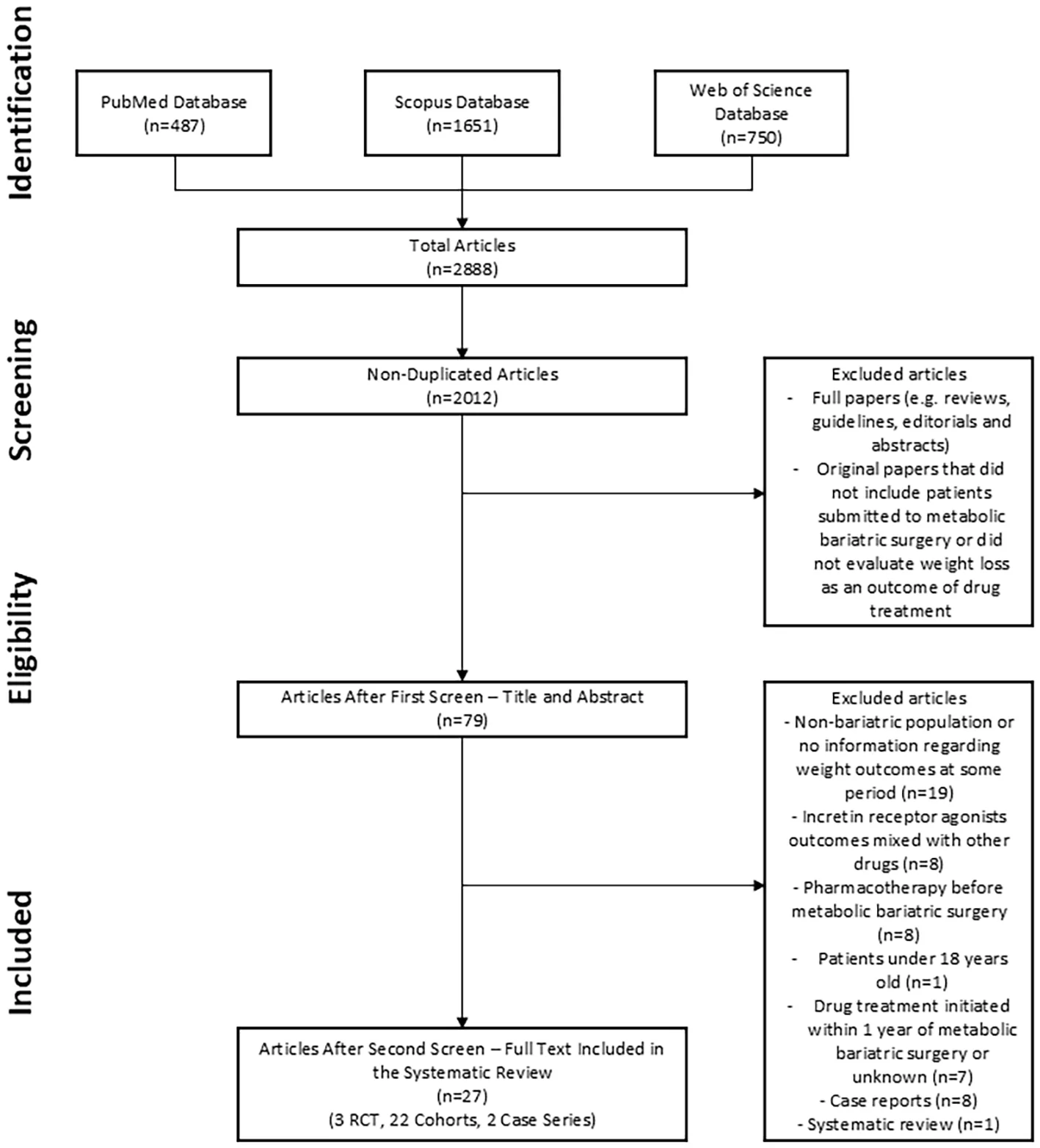
PRISMA Flowchart of the search, eligibility criteria approaches and study inclusion for the systematic review.

**Figure 2:**
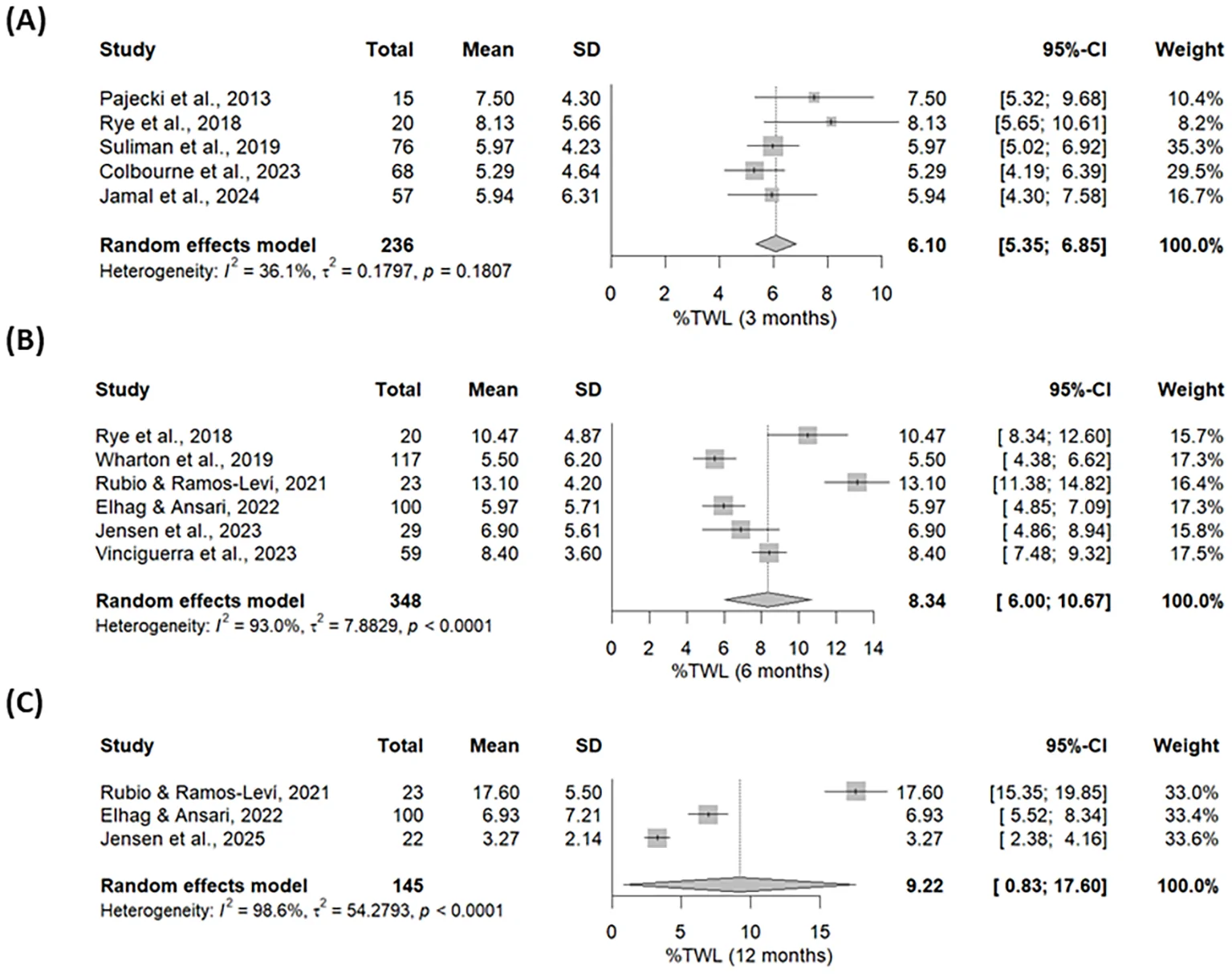
Forest plots for the percentage of total weight loss (%TWL) with the use of liraglutide after a treatment period of 3 months **(A)**, 6 months **(B)**, and 12 months **(C)**.

**Figure 3:**
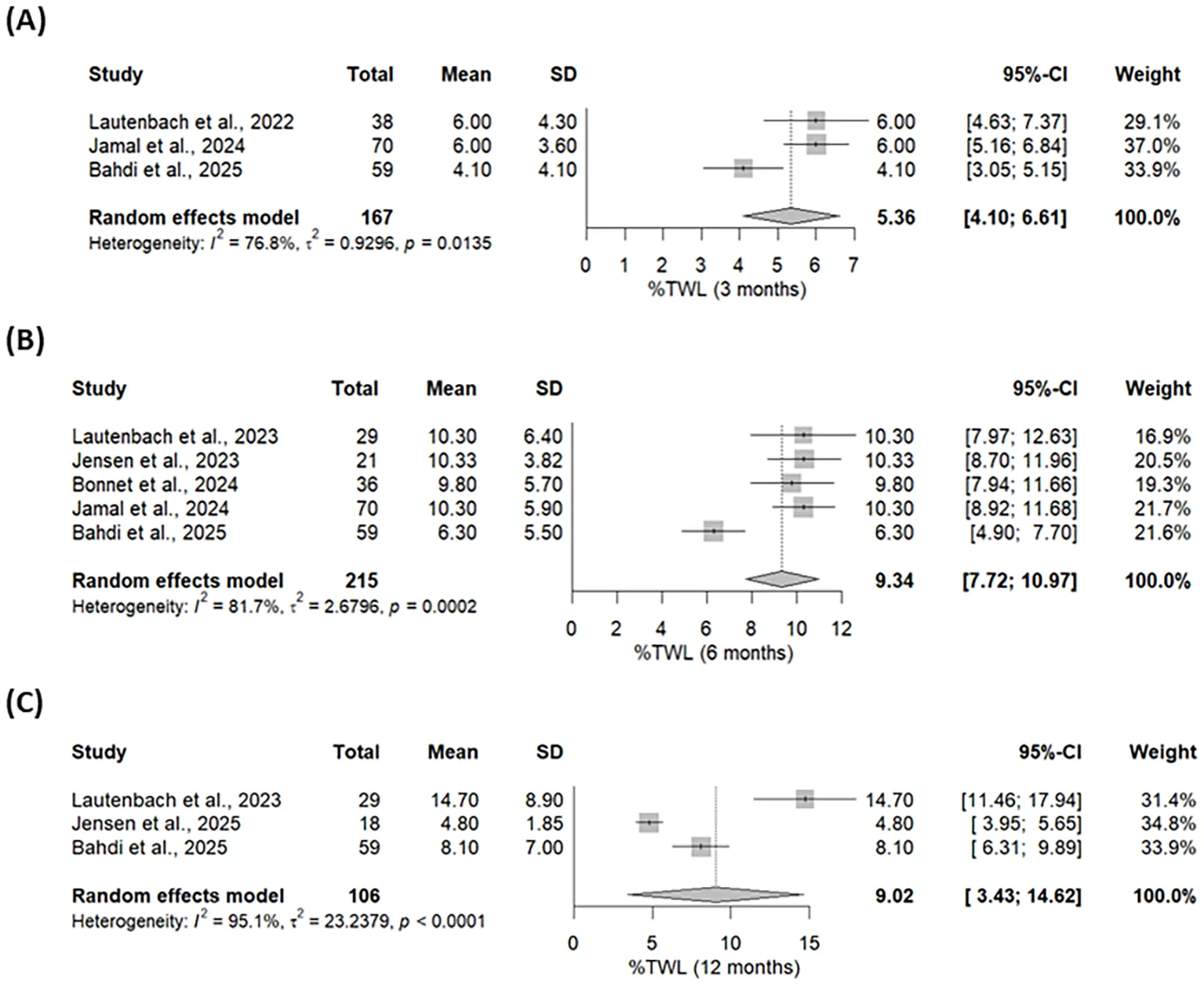
Forest plots for the percentage of total weight loss (%TWL) with the use of semaglutide after a treatment period of 3 months **(A)**, 6 months **(B)**, and 12 months **(C)**.

The search identified 2888 papers (487 from PubMed, 1651 from Scopus, and 750 from Web of Science). After removal of 876 duplicates, a total of 2012 papers were submitted for screening. By reading the titles and abstracts, 1933 papers were excluded, resulting in 79 papers to be considered for inclusion. A second screening to assess eligibility was performed after reading the full paper, which resulted in 52 additional papers being excluded. This resulted in 27 papers to be included in the systematic review (**Figure 1**) – 3 randomized controlled trials, 22 cohort studies, and 2 case series (**Supplementary File 2**). All papers included passed the quality assessment, and the scores are described in **Supplementary File 3**.

### 3.2 Characteristics of the studies

The studies selected evaluated the use of liraglutide (n=15) (22–36), semaglutide (n=4) (37–40), tirzepatide (n=1) (41), and the comparison between different incretin receptor agonist drugs (n=7) (42–48). Studies were performed in different countries: Australia (34), Brazil (30), Canada (27, 28), France (39), Germany (37, 38, 41), Italy (23, 35), Japan (40), Kuwait (32, 43), Spain (25, 29), Switzerland (24, 45, 46), United Arab Emirates (36), United Kingdom (26, 33), United States of America (31, 42, 44, 47, 48), and Qatar (22). Study participants had an age range spanning from 18 to over 60 years old, with a predominance of female individuals. The majority of patients have undergone either Roux-en Y gastric bypass (RYGB) or sleeve gastrectomy (SG). **Table 1** summarizes the outcomes of drug treatment encompassing weight trajectories and WL results, including the dosage used, sample size of each study, time since surgery until drug treatment initiation, treatment duration for each study, and the definition for WR and/or IWL of each study, more detailed information can be found in **Supplementary File 2**. However, it should be stressed that for Jamal et al. (43), the duration of treatment was not specified.

**Table 1:** Summary of weight loss outcomes of incretin receptor agonists treatment in subjects submitted metabolic bariatric surgery.

First author and year	N	Weight status before treatment	Time since MBS	IWL or WR definition	Dosage	Months	Weight loss outcomes
Elhag & El Ansari, 2022 (22)	119	NR	56.47 ± 32.29 months	WR: >10kg from nadir IWL: <50% of excess weight lost at 18 months after intervention	Liraglutide – 3 mg	6	Body weight of 91.01 ± 17.30 kg and %TWL of 5.97%
						12	Body weight 88.44 ± 15.47 kg and %TWL of 6.93%
Rubio & Ramos-Leví, 2021 (25)	23	BMI of 36.6 ± 4.6 kg/m^2^	72 months	WR: >15% from nadir	Liraglutide – 3 mg	6	BMI of 31.8 ± 4.2 kg/m^2^ and %TWL of 13.1%
						12	BMI of 30.1 ± 4.0 kg/m^2^ and %TWL of 17.6%
Wharton et al., 2019 (27)	117	BMI of 42.5 ± 9.6 kg/m^2^	96 months	WR: + 10-25% of excess weight or total weight lost	Liraglutide – 3 mg	7.6	%TWL of 5.5%
Rye et al., 2018 (28)	20	Body weight of 117.9 ± 27.95 kg *	76.3 ± 72.9 months	WR: >10% from nadir IWL: <20% WL from baseline	Liraglutide – 2.9 mg	± 3.75	Body weight of 108.9 ± 28.10 kg* and %TWL of 7.1% (IQR 5.1–12.2%)
						± 6.5	Body weight of 105.6 ± 27.49 kg* and %TWL of 9.7% (IQR 7.8–13.9%)
Pajecki et al., 2013 (30)	15	Body weight of 100.9 ± 18.3 kg	63.6 months	WR: >15% from nadir IWL: <50% of excess weight	Liraglutide – 1.8 mg	± 3	Body weight of 93.5 ± 17.4 kg and %TWL of 7.38%
Lofton et al., 2025 (31)	58	NR	18–120 months	WR: >10% from nadir	Liraglutide – 3 mg	± 12.8	%TBWL of -8.8%
Jamal et al., 2024 (32)	57	Body weight of 96.12 ± 29.26 kg	± 120 months	WR: ≥10% from the lowest post-SG weight IWL: <20% WL from preoperative weight	Liraglutide – 3 mg	3	Body weight of 90.19 ± 26.82 kg and %WL of 6.20%
Vinciguerra et al., 2023 (35)	59	BMI of 38.2 ± 5.7 kg/m^2^	20 ± 6 months	WR: gain of at least 15% of the WL after metabolic bariatric surgery IWL: initial WL <50% of EWL	Liraglutide – 2.4 mg	± 5.5	BMI of 35.1 ± 5.6 kg/m^2^ and %WL of 8.4%
Suliman et al., 2019 (36)	189	NR	± 48 months	ND	Liraglutide – 3 mg	± 3.6	%WL of 5.6% for RYGB patients and 3.3% for SG patients
Colbourne et al., 2023 (34)	68	Median body weight of 95.8 (93.4-117.6) kg	At least 12 months after LSG and RYGB and at least 24 months after LGB	IWL: BMI> 35 kg/m2 (all patients), >25% TWL (LSG; RYGBP), >20% TWL (LAGB)	Liraglutide – 1.8 to 3 mg	3	Median body weight of 90.3 (78.5-113.1) kg and %TBWL of 5.30%
Jamal et al., 2024 (43)	70	Body weight of 90.1 ± 19.4 kg	71.8 ± 51.1 months	WR: *a priori* ≥ 10% weight recurrence from the nadir post-SG weight	Semaglutide	3	Body weight of 94.9 ± 19.4 kg and %WL of 6.0%
						6	Body weight of 81.0 ± 29.0 kg and %WL of 10.3
	45	Body weight of 100.2 ± 28.5 kg			Tirzepatide	3	Body weight of 91.2 ± 27.3 and %WL of 9.3
						6	Body weight of 87.6 ± 28.3 kg and %WL of 15.5
Bahdi et al., 2025 (44)	59	NR	52.4 ± 33.8 months	Weight recidivism: ≥25% weight gain from nadir weight following SG	Semaglutide – 1.25 mg	3	%TBWL of 4.1%
						6	%TBWL of 6.3%
						12	%TBWL of 8.1%
	7				Tirzepatide – 7.5 mg	3	%TBWL of 3.6%
						6	%TBWL of 13.5%
						12	%TBWL of 13.2%
Jensen et al., 2023 (45)	29	Body weight of 90.5 (83.4, 107.9) kg	72 months	WR: any weight gain following the weight nadir at least 12 months after BS	Liraglutide – 3 mg	6	%TBWL of 7.3%
	21				Semaglutide – 1 mg		%TBWL of 9.8%
Lautenbach et al., 2022 (37)	44	Body weight of 113.5 ± 25.2 kg	64.7 ± 47.6 months	WR: continuous WR after an initial successful WL (defined as EWL>50%) IWL: achieving a nadir weight with EWL<50% after surgery	Semaglutide – 0.5 mg	3	Body weight of 106.5 ± 24.5 kg and %TWL of -6.0
						6	Body weight of 105.7 ± 25.1 kg and %TWL of -10.3
Lautenbach et al., 2023 (38)	29	Body weight of 110.8 ± 22.2 kg	64.5 ± 51.9 months	WR: continuous WR after an initial successful WL (defined as EWL>50%) IWL: achieving a nadir weight with EWL<50% after surgery	Semaglutide – 1mg	6	Body weight of 99.2 ± 22.9 kg and %TWL of 10.3%
						12	Body weight of 95.4 ± 24.0 kg and %TWL of 14.7%
Bonnet et al., 2024 (39)	36	Body weight of 125.2 ± 23.6 kg	100.8 months	WR IWL: EWL<50% 18 months post-BS	Semaglutide – 2.4 mg	± 5.5	%WL of - 9.8%
Stoll et al., 2025 (41)	21	Body weight of 122.1 ± 24.8 kg	86.4 ± 51.6 months	WR: continuous weight increase after an initial successful WL (EWL>50% 18 months post-MBS) IWL: achieving a nadir weight with EWL <50% 18 months after surgery	Tirzepatide – 5mg	6	%TWL of 12%

*Calculated percentage. BMI, body mass index; EWL, excess weight loss; IQR, interquartile range; LAGB, laparoscopic gastric banding; LSG, laparoscopic sleeve gastrectomy; MBS, metabolic bariatric surgery; N, number of subjects submitted to liraglutide treatment; NR, not reported in source study; ND, not determined; RYGB, Roux-en-Y gastric bypass; SG, sleeve gastrectomy; %TBWL, percentage of total body weight loss; %TWL, percentage of total weight loss; %WL, percentage of weight loss; WR, weight regain.

### 3.3 Weight loss effectiveness: a descriptive approach

#### 3.3.1 Liraglutide

Fifteen studies (n=15) evaluated the effectiveness of liraglutide treatment in patients previously submitted to metabolic bariatric surgery (22–36). From those, ten papers (n=10) (22, 24, 25, 29–35) aimed to evaluate the WL effectiveness of liraglutide in post-bariatric patients with WR and/or IWL. The criteria used to define WR or IWL varied, with all studies using different definitions. WR was defined by regaining more than 5 to 25% of body weight from the post-intervention body weight nadir. IWL was defined as losing less than 20 to 50% of the total body weight/excess weight, or not achieving a body mass index under 35 kg/m^2^.

For meta-analysis, data were divided according to the treatment period: 3 months (n=5) (28, 30, 32, 34, 36), 6 months (n=6) (22, 25, 27, 28, 35, 45), and 12 months (n=3) (22, 25, 46) (**Figure 2**). The analysis showed a %TWL of 6.10% at 3 months (95% CI 5.35 to 6.85), of 8.34% at 6 months (95% CI 6.00 to 10.67), and of 9.22% at 12 months (95% CI 0.83 to 17.60). Heterogeneity was detected in terms of %TWL among the studies analyzed at 3 months (I^2^ = 36.1%, τ^2^ = 0.1797, p=0.1807), at 6 months (I^2^ = 93.0%, τ^2^ = 7.8829, p<0.0001), and at 12 months (I^2^ = 98.6%, τ^2^ = 54.2793, p<0.0001).

In addition to the meta-analysis, other relevant findings are described, as these provide important contextual information. It is worth noting that randomized controlled trials were not included in the meta-analysis as they substantially differed from the other studies included, namely in study design, patient selection, as well as range of results, leading to heterogeneity that precluded the quantitative pooling. The randomized controlled trials at approximately 6 months with liraglutide were a study by Mok et al., in which patients presented a percentage of body weight change of -8.82% (33), and a study by Miras et al., with an average weight change difference of -2.34 kg (26), whereas at 12 months the only study was by Lofton et al., in which an average percentage of total body weight change of -8.8% was achieved (31). Further, Muratori et al. (23) and Wharton et al. (27) reported that the WL efficacy of liraglutide was not influenced by the type of metabolic bariatric surgery (laparoscopic sleeve gastrectomy (LSG), laparoscopic gastric banding (LGB), RYGB, and SG). Rubio and Ramos-Leví evaluated the WL effectiveness of liraglutide in patients with WR after metabolic bariatric surgery in two groups, as they tested two treatment protocols, namely daily treatment and alternate-day treatment. Authors concluded that every other day dosing was as effective as daily dosing after 6 months of treatment, with more than 85% of the patients losing the weight regained (25). Furthermore, a prospective cohort study divided patients into 4 groups – lifestyle intervention, liraglutide treatment, revisional endosurgery and Fobi-ring with pouch resizing surgery. Grounded on the study results, the authors suggested that liraglutide should be recommended as a first-line therapy for WR after RYGB, and revision surgery should be reserved for non-responders or patients in whom liraglutide is contraindicated (24). Of note, Elhag & El Ansari concluded that liraglutide was equally effective for WR and IWL in patients submitted to primary and revisional surgeries (22).

#### 3.3.2 Semaglutide

Four articles (n=4) evaluated the effectiveness of semaglutide in patients previously submitted to metabolic bariatric surgery (37–40). All four papers aimed to assess the efficacy of semaglutide in post-bariatric patients with WR and/or IWL. The criteria used to define WR or IWL also varied. WR was defined as continuous WR after an initial WL or weight recurrence of 5% from nadir. IWL was defined as achieving a nadir weight with an excess weight loss inferior to 50%.

A meta-analysis of studies reporting WL outcomes after treatment with semaglutide was conducted, with data stratified according to treatment duration: 3 months (n=3) (37, 43, 44), 6 months (n=5) (38, 39, 43–45), and 12 months (n=3) (38, 44, 46) (**Figure 3**). It is worth noting that for the meta-analysis involving the 3 months period, only Lautenbach et al., (37) was included, whereas for the 6 months, only Lautenbach et al., (38) was included. The analysis showed a %TWL of 5.36% at 3 months (95% CI 4.10 to 6.61), of 9.34% at 6 months (95% CI 7.72 to 10.97), and of 9.02% at 12 months (95% CI 3.43 to 14.62). Heterogeneity was disclosed regarding %TWL among the studies analyzed at 3 months (I^2^ = 76.8%, τ^2^ = 0.9296, p=0.0135), at 6 months (I^2^ = 81.7%, τ^2^ = 2.6796, p=0.0002), and at 12 months (I^2^ = 95.1%, τ^2^ = 23.2379, p<0.0001).

Additionally to the meta-analysis, Kanai et al. (40) assessed the effects of treatment with semaglutide 1.0 mg weekly in individuals previously submitted to LSG. One year after treatment, mean body weight decreased from 104.6 kg to 100 kg, whereas BMI reduced 1.6 kg/m^2^.

#### 3.3.3 Tirzepatide

One article (n=1) assessed the WL effectiveness of 5.0 mg weekly tirzepatide treatment for 6 months in patients with IWL or WR after RYGB and SG (41). Tirzepatide treatment led to a %TWL of 12%, with all patients achieving a WL equal to or greater than 5%.

#### 3.3.4 Comparison between different incretin receptor agonists

**Figure 4:**
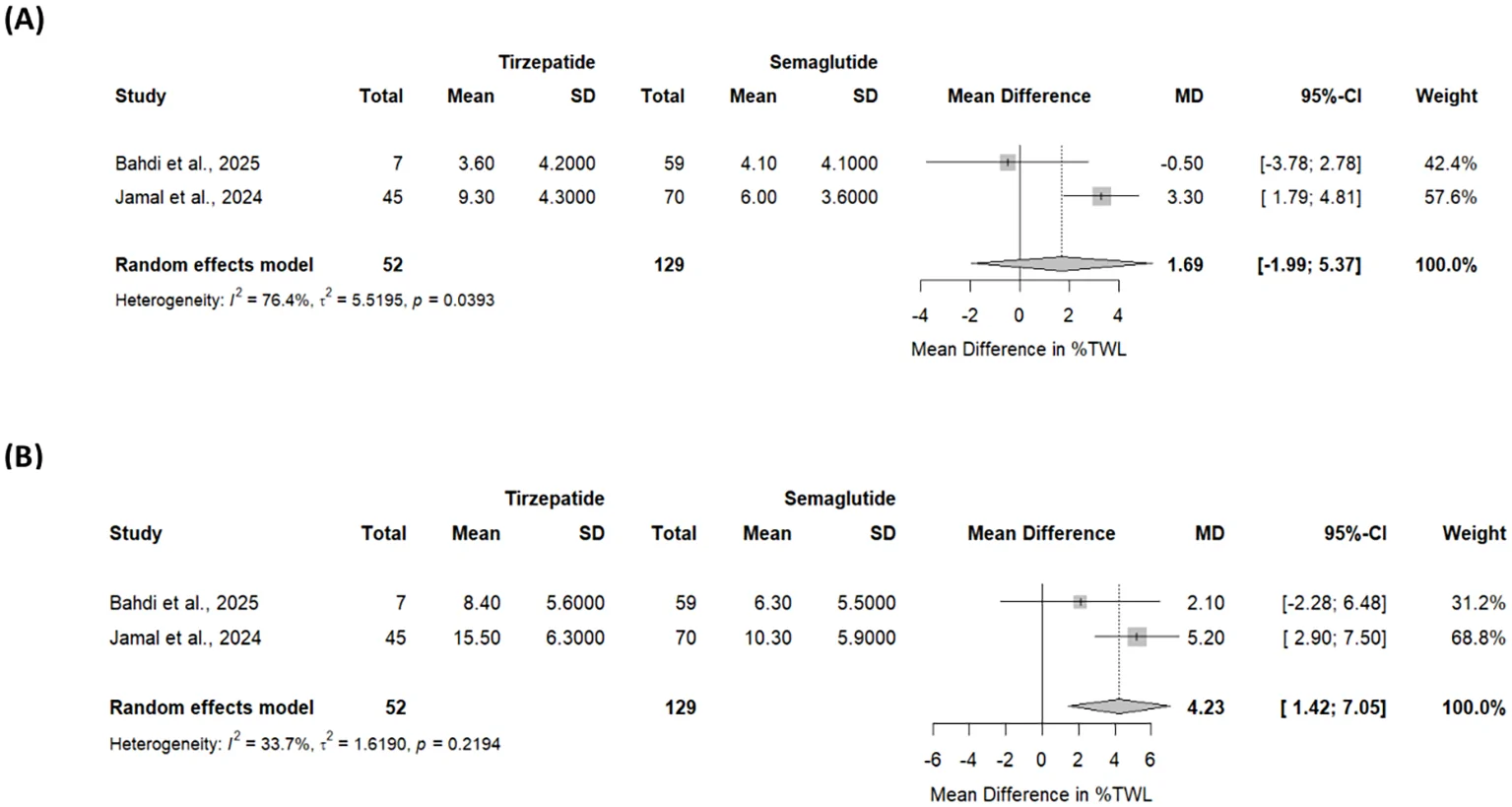
Forest plots for the mean difference of the percentage of total weight loss (%TWL) comparing the use of tirzepatide and semaglutide after a treatment period of 3 months **(A)** and 6 months **(B)**.

Seven articles (n=7) compared the effectiveness and safety of incretin receptor agonist drugs in patients previously submitted to metabolic bariatric surgery (42–48). Of those seven articles, two were used for the meta-analysis to compare semaglutide and tirzepatide (43, 44) (**Figure 4**). In the study by Jamal et al., 68.6% of the patients were treated with a semaglutide dosage equal to or superior to 1 mg and 63.8% received a tirzepatide dosage equal to or superior to 10 mg (43). In Bahdi et al., the therapeutic dosage for semaglutide was 1.25 mg and for tirzepatide was 7.5 mg (44). The data analysis revealed a mean difference in %TWL of 1.69% at 3 months (95% CI -1.99 to 5.37) and of 4.23% at 6 months (95% CI 1.42 to 7.05), in favor of tirzepatide. Significant heterogeneity was observed among studies at 6 months (I^2^ = 76.4%, τ^2^ = 5.5195, p=0.0393), whereas at 12 months heterogeneity was lower (I^2^ = 33.7%, τ^2^ = 1.6190, p=0.2194).

Additionally, Stanford et al. evaluated the effectiveness of fifteen obesity management medications in patients previously submitted to RYGB or SG (42), in which only liraglutide achieved a significant negative beta coefficient in the linear regression analysis as a WL predictor. Moreover, a study by Firkins et al. analyzed the use of obesity medications (48), with significant differences found for the number of individuals with BMI>30kg/m^2^ and >40kg/m^2^, both in the semaglutide and liraglutide groups. Three studies compared the use of liraglutide and semaglutide in patients with WR after metabolic bariatric surgery, in which the semaglutide group exhibited greater WL outcomes when compared to the liraglutide group (45–47).

It is worth noting that a high heterogeneity level was present for most of the meta-analyses performed. This can be attributed to the diversity in the population’s characteristics, types of metabolic bariatric surgery procedures performed, WL evaluation, time span since metabolic bariatric surgery, and drug dosage diversity across all the studies.

### 3.4 Adverse events associated with incretin receptor agonist treatment

No serious adverse events related to drug treatment were reported. Most studies reported the occurrence of non-serious adverse events similar to those described in clinical trials, which mostly included mild gastrointestinal symptoms. The percentage of adverse events reported by each study is summarized in **Table 2**.

**Table 2:** Summary of adverse events reported to be associated with incretin receptor agonist treatment.

First author and year	Total adverse event rate	Specific adverse events rate	Discontinuation rate
Elhag & El Ansari, 2022 (22)	15.4%	10.8% nausea; 15% constipation, diarrhea, fatigue, headache, and indigestion; 3.1% bloating,vomiting	NR
Muratori et al., 2022 (23)	11.3% *	11.3% nausea and vomiting in the beginning *	NR
Horber & Steffen, 2021 (24)	NR	Nausea was common in the first weeks of treatment and gastrointestinal effects resulted in not reaching a 3.0 mg dose	NR
Rubio & Ramos-Leví, 2021 (25)	65.2%	65.2% mild and transient gastrointestinal malaise during the first weeks	NR
Miras et al., 2019 (26)	45%	17% nausea, 2% diarrhea, 8% constipation, 4% abdominal discomfort, 2% gastro-oesophageal reflux, 2% headache, 2% injection-site bruising	NR
Wharton et al., 2019 (27)	50.4%	29.1% nausea, 11.1% constipation, 6.8% diarrhea, 6% fatigue, 3.4% headache, 3.4% rash, 2.6% indigestion, 2.6% vomiting, 2.6% dry mouth, 1.7% bloating, 1.7% sweating, 7.7% others, which included abdominal pain, bruising, decreased glomerular filtration rate, depression, flu-like symptoms, heartburn, hot flashes, gas, pancreatitis	NR
Rye et al., 2018 (28)	NR	37.9 % nausea; 37.9% headache; 34.5% gastroesophageal reflux; 31% constipation, taste dysgeusia, diarrhea; 13.8% vomiting, dizziness; 10.3% fatigue; 27.6% with other, which included one patient for each of the following adverse events: epigastric pain, umbilical cellulitis, belching, esophageal dysphagia, profound anorexia, rash, back spasm	NR
Gorgojo-Martínez et al., 2016 (29)	NR	No severe GI adverse reactions leading to withdrawals and one patient with mild transient event, nausea and vomiting (6.7%)	NR
Pajecki et al., 2013 (30)	NR	40% nausea	NR
Lofton et al., 2025 (31)	NR	24.72% nausea, 15.73% constipation, 10.11% abdominal pain, 7.87% fatigue, 4.49% diarrhea, 4.49% dry mouth, 6.74% decreased appetite	NR
Jamal et al., 2024 (32)	NR	NR	NR
Vinciguerra et al., 2023 (35)	NR	Mild nausea registered at the beginning of the treatment	NR
Suliman et al., 2019 (36)	NR	Mild to moderate gastrointestinal symptoms, including nausea, vomiting, diarrhea	NR
Mok et al., 2023 (33)	80%	51% nausea, 6% diarrhea, 26% constipation, 3% vomiting, 6% abdominal pain, 3% abdominal bloating, 3% dyspepsia, 3% headache	NR
Colbourne et al., 2023 (34)	NR	11.8% reported side effects, which could include one or more effects of unspecified nature, 5.9% abdominal pain, 1.5% constipation, 4.4% nausea, 1.5% palpitations, 1.5% lethargy, 1.5% rash	NR
Stanford et al., 2018 (42)	NR	NR	NR
Jamal et al., 2024 (43)	NR	30% mild adverse events with semaglutide and 34% with tirzepatide. The reported adverse events included nausea, fatigue, vomiting, constipation, diarrhea, hair loss, heartburn, injection site reaction	NR
Bahdi et al., 2025 (44)	NR	Semaglutide - 22% nausea ± vomiting Tirzepatide - 14.3% nausea ± vomiting	18.6% semaglutide 14.3% tirzepatide
Jensen et al., 2023 (45)	36%	22% nausea, 10% obstipation, 2% vomiting, 2% flatulence, 2% diarrhea, 2% headache, 2% dizziness, 2% injection site reaction	NR
Jensen et al., 2025 (46)	32.5%	Semaglutide – 44.4% (22.2% constipation) Liraglutide – 22.7% (4.5% constipation)	NR
Murvelashvili et al., 2023 (47)	NR	NR	NR
Firkins et al., 2024 (48)	NR	NR	NR
Lautenbach et al., 2022 (37)	NR	4.54% nausea, 2.27% increased pancreatic lipase levels *	NR
Lautenbach et al., 2023 (38)	NR	NR	NR
Bonnet et al., 2024 (39)	5.1%	NR	5.1%
Kanai et al., 2024 (40)	NR	Patients with adverse events were excluded	NR
Stoll et al., 2025 (41)	NR	NR	NR

*Calculated percentage of patients that had an adverse event. NR – not reported in source study.

**Figure 5:**
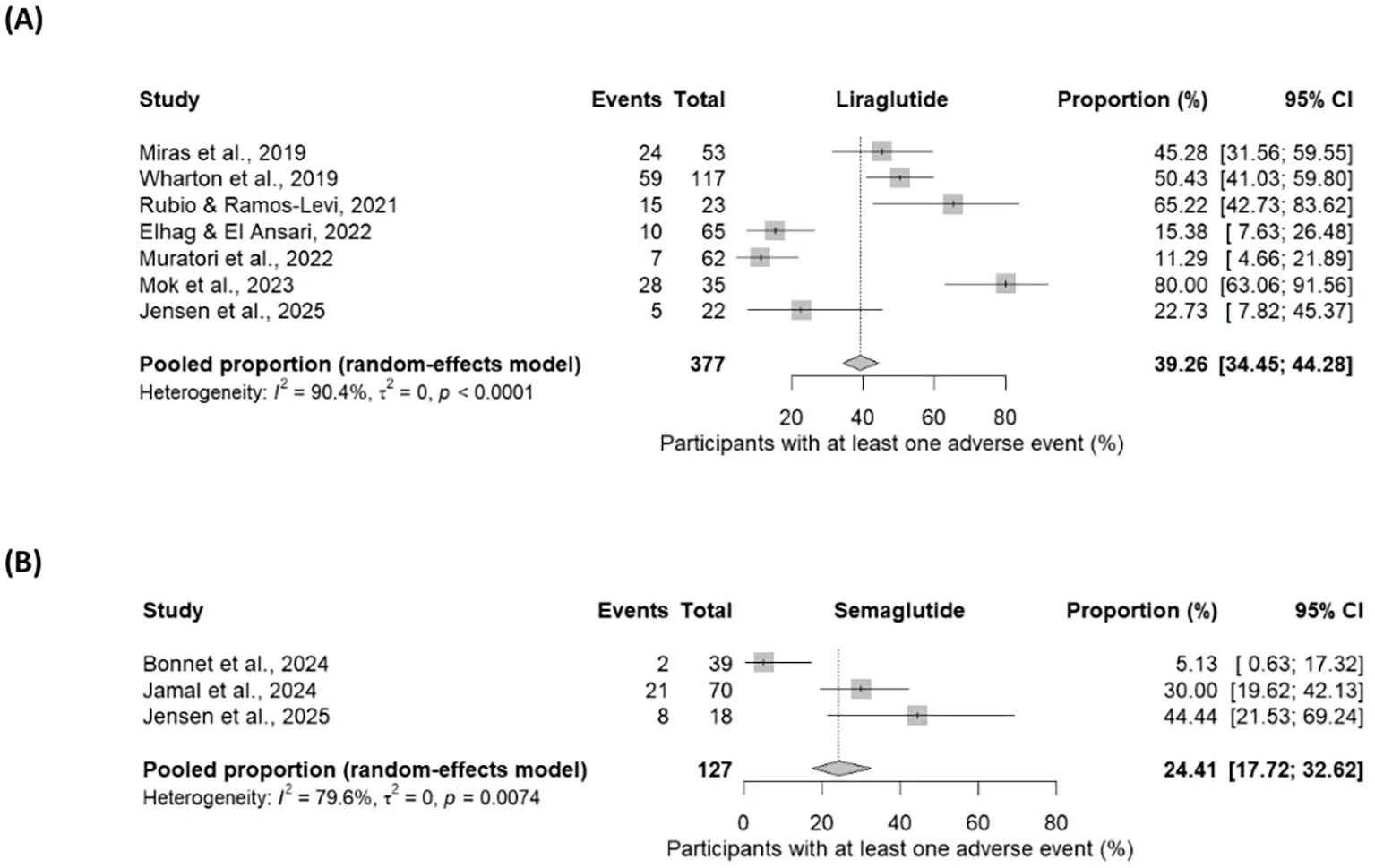
Forest plot for the proportion of participants experiencing at least one adverse event among those receiving liraglutide **(A)** and semaglutide **(B)**.

A meta-analysis of studies reporting adverse events after treatment with incretin receptor agonists was conducted, with data stratified according to the drug used, liraglutide and semaglutide. Regarding the adverse events associated with the use of liraglutide, a symptom-specific meta-analysis was conducted. Seven studies (n=7), including 377 participants treated with liraglutide, reported the proportion of participants experiencing at least one adverse event. The pooled proportion was 39.26% (95% CI 34.45 to 44.28%), with substantial between-study heterogeneity (I^2^ = 90.4%, τ^2^ = 0, p<0.0001) (**Figure 5**). In the symptom-specific analyses, nausea was the most frequently reported adverse event, with a pooled proportion of 23.16% (95% CI 19.58 to 27.17%; 8 studies; n = 475). This was followed by constipation (10.79%, 95% CI 8.31 to 13.89%; 8 studies; n = 482), diarrhea (6.38%, 95% CI 4.35 to 9.27%; 6 studies; n = 392), headache (6.38%, 95% CI 4.35 to 9.27%; 6 studies; n = 392), fatigue (5.92%, 95% CI 3.76 to 9.20%; 4 studies; n = 304), abdominal pain or discomfort (4.97%, 95% CI 3.15 to 7.75%; 5 studies; n = 362), and vomiting (4.00%, 95% CI 2.17 to 7.27%; 4 studies; n = 250) (**Supplementary Figure 1**). The number of studies and participants varied across symptom-specific analyses given that only studies reporting each adverse event separately were included. Studies reporting combined outcomes, such as nausea and vomiting, were excluded from the corresponding symptom-specific analyses. For semaglutide, three studies (n=3) involving 127 participants reported the proportion of participants experiencing at least one adverse event. The pooled proportion was 24.41% (95% CI 17.72 to 32.62%), with substantial between-study heterogeneity (I^2^ = 79.6%, τ^2^ = 0, p=0.0074) (**Figure 5**). Despite the high I^2^, τ^2^ was estimated as 0, which may reflect the imprecision of between-study variance estimation when only three studies are available.

## 4 Discussion

This systematic review aimed to compile the best available evidence on the use of incretin receptor agonist drugs in patients who have undergone metabolic bariatric surgery, including the use for IWL and WR. Ultimately, addressing a knowledge gap and unmet need, as the use of these drugs in this population remains limited in clinical trials.

Inadequate maintenance of the WL achieved or a suboptimal clinical response to metabolic bariatric surgery requires further intervention to treat obesity and related complications. Conservative approaches for these cases include lifestyle interventions and/or pharmacological treatments as appropriate (49). Revision surgery can be considered, which eventually occurs in some patients. However, given the increased risk of short and long-term complications, surgery should only be considered when other non-invasive options have proven to be ineffective (50, 51).

The studies included in this review have shown that treatment with incretin receptor agonist drugs in patients previously submitted to metabolic bariatric surgery resulted in significant WL, with tirzepatide showing greater outcomes, suggesting their potential as a rescue therapy after metabolic bariatric surgery. The wide discrepancies in WL between studies could be attributed to unprecedented differences in patient populations, type of metabolic bariatric surgery performed, timing of treatment initiation, drug dosage, and treatment duration.

In randomized controlled trials, patients with obesity, excluding individuals with previous metabolic bariatric surgery, treated with this class of drugs were shown to achieve an average WL ranging from 5% to 20%. Therefore, the range of percentage of WL identified by this systematic review is consistent with previous data, suggesting that the efficacy of incretin receptor agonist treatment in post-bariatric patients is likely comparable to that observed in the general population with obesity. Noticeably, the study with the best WL outcome of liraglutide treatment, achieving a %TWL of 17.6%, initiated treatment 72 months after surgery (25). When it comes to semaglutide, the greatest outcome was 14.7% of %TWL, with treatment being initiated approximately 64.5 months after metabolic bariatric surgery (38). Both of these studies had a treatment duration of 1 year. Regarding tirzepatide, the most significant outcome was achieved after 6 months, with an average WL of 15.5%, with treatment initiation 71.8 months after metabolic bariatric surgery. It is worth noting that when initiating a treatment with an incretin receptor agonist two years or more after metabolic bariatric surgery, the WL results depend predominantly on the drug treatment, since a phase of stable weight is likely to have been reached by that time.

Additionally, our meta-analysis demonstrated that WL outcomes with an incretin receptor agonist after metabolic bariatric surgery are similar with those reported in clinical trials. With respect to liraglutide, a %TWL of 6.10%, 8.34%, and 9.22% were observed for treatment durations of 3, 6, and 12 months, respectively. Moreover, the longer the treatment duration, the greater the WL outcomes were. This finding was also reported in a study by Davies et al., involving patients without prior metabolic bariatric surgery, with a mean WL of 6.0% being observed after 56 weeks of liraglutide treatment (52), and in a study by Farías et al., in which patients who had previously undergone metabolic bariatric surgery achieved a mean %WL of 7.6% at 12 months (53). For semaglutide, our meta-analysis revealed that patients submitted to metabolic bariatric surgery had a %TWL of 5.36%, 9.34%, and 9.02%, after 3, 6, and 12 months of treatment, respectively. Further, it also demonstrated that a longer treatment duration was associated with a greater WL. This is in accordance with the studies by Garvey et al., in which patients, without prior metabolic bariatric surgery, achieved a mean body weight change of -15.2% at 104 weeks after semaglutide treatment (54). Lastly, our meta-analysis revealed a mean difference in %TWL of 4.23% after 6 months of treatment with tirzepatide when compared to semaglutide. This is in agreement with the findings of Rodriguez et al., a study that included a group of individuals previously submitted to metabolic bariatric surgery, which concluded that after 12 months of treatment, tirzepatide achieved a greater weight change than semaglutide (-15.3% vs -8.3%) (15), which was also confirmed at the clinical trial SURMOUNT-5, comparing the WL efficacy of the two drugs after 72 weeks, in patients with obesity and no prior metabolic bariatric surgery (-20.2% vs -13.7%) (55).

Our meta-analysis demonstrated that the pooled proportion of participants experiencing at least one adverse event was 39.26% among those receiving liraglutide and 24.41% among those receiving semaglutide. These estimates should not be interpreted as a direct comparison between treatments given that they were derived from different studies and populations. In the symptom-specific analyses of liraglutide, nausea was the most frequently reported event (23.16%), followed by constipation (10.79%), diarrhea (6.38%), headache (6.38%), fatigue (5.92%), abdominal pain or discomfort (4.97%), and vomiting (4.00%). Noteworthy, no serious adverse events attributed to treatment were reported in this patient population. Overall, the reported safety profile was predominantly characterized by mild gastrointestinal symptoms, consistent with evidence from clinical trials conducted in non-bariatric populations (52, 56, 57). Patients should therefore be informed about the likelihood of gastrointestinal symptoms when initiating therapy. Nevertheless, these findings should be interpreted with caution since adverse-event reporting was incomplete, as only a limited number of studies contributed to each analysis, and symptom-specific meta-analyses could not be performed for semaglutide.

Given the rapid development of this field, an updated compilation of the best available and most recent evidence was warranted. Previously, available systematic reviews addressed the efficacy and safety of incretin receptor agonists in patients who underwent metabolic bariatric surgery and reported greater WL outcomes with semaglutide than with liraglutide. More recently, evidence also suggests that tirzepatide may produce even greater WL results (17–19). Compared to the above mentioned reviews, the herein systematic review by including a greater number of studies and conducting a meta-analysis builds-on and expands the available evidence on the topic. Additionally, it incorporates novel evidence on tirzepatide in this specific population and encompasses a meta-analysis comparing treatment with tirzepatide and semaglutide. A comparison that was not conducted by previous systematic reviews and meta-analysis. Moreover, our work provides a quantitative analysis of adverse events associated with incretin-based therapies in this patient population.

Despite addressing a relevant knowledge gap, this systematic review and meta-analysis hold a few limitations that must be acknowledged. One limitation is the lack of consistency across studies in the definitions of “suboptimal initial response” and “recurrent weight gain” following metabolic bariatric surgery. This issue is widely acknowledged in the literature and has a broad impact on the evaluation and comparison of metabolic bariatric surgery outcomes (6, 7). This limitation also poses challenges for assessing post-surgical outcomes and for standardizing emerging obesity interventions, whether surgical or non-surgical. Additionally, as a consequence of different criteria used to define suboptimal clinical responses across studies, the patient populations will inevitably present distinctive phenotypic features, which is another limitation found when compiling the data from different studies. In fact, by restricting the inclusion criteria to studies that evaluated the efficacy and safety of these drugs in patients with at least one year after metabolic bariatric surgery who had experienced a suboptimal clinical response, this review has drastically limited the number of eligible studies and sample size of patients included, and therefore also precluded the possibility of conducting subgroup analyses. Another limitation was the variety of metrics used for reporting WL outcomes, with the most common being absolute WL and %TWL, although BMI change and percentage of excess weight loss (%EWL) were also used as the sole measures of WL outcomes, which made it impossible to establish meaningful comparisons, particularly for the meta-analysis. Indeed, the lack of non-computed anthropometric data, such as body weight before and after treatment initiation, precluded further comparisons and interpretation of the results, especially the inclusion of long-term outcomes. As a matter of fact, a prior call to action urged researchers to consistently report body weight and BMI to facilitate data aggregation and the generation of more robust real-world evidence (58). Concurrently, a high heterogeneity was observed for the majority of the meta-analyses performed. There are several possible reasons for this heterogeneity, including diversity of the population’s characteristics, types of metabolic bariatric surgery performed, WL evaluation, time span since metabolic bariatric surgery, and drug dosage diversity across all the studies. Given the heterogeneity in dosage, follow-up duration and study design, the pooled estimates of adverse events should be interpreted cautiously. Concurrently, publication bias analyses and sensitivity analyses were not performed due to the small number of studies that contributed to each meta-analysis. However, the possibility of publication bias or other reporting biases cannot be excluded. The discontinuation of therapy is also an uncharted territory, since in the majority of the studies, there are no reports on weight trajectories of post-bariatric patients after treatment withdrawal. Given that obesity is a chronic disease requiring long-term therapy, treatment discontinuation is commonly associated with increased appetite, reduced satiety, and subsequent weight regain, along with the recurrence of obesity-associated disorders (59). Finally, although the WL effectiveness of incretin receptor agonist therapy has been demonstrated, cost-benefit considerations should also be taken into account, as it can influence the decision to initiate treatment as well as drug discontinuation. However, the absence of financial cost-effectiveness analyses in the available studies prevented the inclusion of this variable in this review.

Notwithstanding the aforementioned limitations, this systematic review also holds the major strength of having compiled the best available and most recent evidence on the use of incretin receptor agonist drugs in post-bariatric patients, particularly their effectiveness in WL outcomes, as well as their safety. This could be useful to propose an algorithm, expert opinion-based pending prospective validation, for addressing the maintenance of WL achieved and the management of a suboptimal clinical response after metabolic bariatric surgery, taking into consideration the predicted WL outcomes, thus maximizing the probabilities of success while managing patient expectations. Since data showed that the %TWL achieved by an incretin receptor agonist drug could reach approximately 20%, a reasonable algorithm to manage suboptimal clinical responses could be lifestyle interventions for a TWL<5%, adding an incretin receptor agonist drug as add on to lifestyle interventions when a TWL ranging from 5 to 20% is the goal, and consider revision surgery whenever the target is TWL >20%. Nevertheless, the need to revise the herein proposed algorithm for managing suboptimal clinical responses is foreseeable, as novel gut hormone analogues for obesity treatment currently under development enter the market, as well as if novel evidence becomes available from clinical trials conducted in post-bariatric patients, since although the bariatric population has consistently been excluded from the past industry-led randomized clinical trials, this practice is likely to change in the future to come.

Despite these findings, further research is needed to evaluate the use of incretin receptor agonists in patients previously submitted to metabolic bariatric surgery. In particular, we highlight the need for randomized controlled trials to address the effectiveness and safety of these drugs with a focus on individuals with a suboptimal clinical response, namely insufficient weight loss and weight regain. Special relevance should also be given to more recent drugs, like tirzepatide and semaglutide, for which evidence in the post-bariatric populations remains limited. Future research should also address relevant clinical questions. These include establishing criteria for patient selection and determining predictors of treatment response to support clinical decision. Additionally, studies should encompass the long-term impact of these drugs on sustained WL, metabolic effects, and durability of treatment benefits. Further, future studies should consider including cost-effective analysis, given its potential impact on the decision to initiate and discontinue treatment, or even on patient adherence.

## 5 Conclusion

Treatment with incretin receptor agonists, liraglutide, semaglutide, and tirzepatide, in patients previously submitted to metabolic bariatric surgery demonstrated significant weight loss outcomes, emerging as an alternative to revisional surgery, and thus can be considered as a rescue therapy for suboptimal clinical response after metabolic bariatric surgery. Ultimately, the data gathered by this systematic review and the results obtained by the meta-analysis provide useful information to guide the decision-making between interventions for managing body weight in patients after metabolic bariatric surgery. Nonetheless, given the substantial heterogeneity observed for most of the meta-analyses performed, these results should be interpreted cautiously.

## Supplementary material

Supplementary Figure 1 Forest plots for the proportion of participants experiencing a symptom-specific event, including nausea **(A)**, constipation **(B)**, diarrhea **(C)**, headache **(D)**, fatigue **(E)**, abdominal pain/discomfort **(F),** and vomiting **(G)**.
