# Supplementary material for: Use of incretin receptor agonists in patients submitted to metabolic bariatric surgery – a systematic review and meta-analysis
Source: Front Endocrinol (Lausanne). 2026 Aug 26;17:1870008. doi: 10.3389/fendo.2026.1870008 (PMC13553220; doi:10.3389/fendo.2026.1870008)
Supplement: Supplementary file 1 [file SupplementaryFile1.docx]

**Supplementary File 1**

DATABASE SEARCH

PubMed:

(“bariatric surger*” OR “bariatric intervention*” OR “obesity surger*” OR “bariatric procedur*”OR "gastric bypass" OR "biliopancreatic diversion" OR "RYGB" OR "sleeve gastrectomy" OR "BPD" OR "BPD-DS" OR "duodenal switch" OR “Single anastomosis duodeno–ileal bypass” OR “SADI-S” OR “SADI” OR “SIPS” OR “SASI” OR “gastric plication” OR “endosleeve” or “endobarrier” OR “gastric clip*” OR “endoscopic bariatric therap*” OR “EBT” OR “endoscopic sleeve gastroplast*” OR “ESG” OR “Intragastric balloon*” OR “Endoluminal bypass liner*” OR “gastric band*”) AND (“liraglutide” OR “semaglutide” OR “Tirzepatide” OR “glucagon-like peptide 1 receptor agonist*” OR “glucagon-like peptide-1 receptor agonist*” OR “GLP-1 receptor agonist*” OR “GLP1 receptor agonist*” OR “GLP-1R agonist*” OR “GLP1R agonist*” OR “GLP-1 analogue” OR “GLP1 analogue”) NOT (“infant*” OR “child*” OR “gestational” OR “pregnanc*” OR “animal*” OR “rat” OR “rats” OR “mice” OR “mouse”)

Scopus:

TITLE-ABS-KEY (“bariatric surger*” OR “bariatric intervention*” OR “obesity surger*” OR “bariatric procedur*”OR "gastric bypass" OR "biliopancreatic diversion" OR "RYGB" OR "sleeve gastrectomy" OR "BPD" OR "BPD-DS" OR "duodenal switch" OR “Single anastomosis duodeno–ileal bypass” OR “SADI-S” OR “SADI” OR “SIPS” OR “SASI” OR “gastric plication” OR “endosleeve” or “endobarrier” OR “gastric clip*” OR “endoscopic bariatric therap*” OR “EBT” OR “endoscopic sleeve gastroplast*” OR “ESG” OR “Intragastric balloon*” OR “Endoluminal bypass liner*” OR “gastric band*”)

AND TITLE-ABS-KEY (“liraglutide” OR “semaglutide” OR “Tirzepatide” OR “glucagon-like peptide 1 receptor agonist*” OR “glucagon-like peptide-1 receptor agonist*” OR “GLP-1 receptor agonist*” OR “GLP1 receptor agonist*” OR “GLP-1R agonist*” OR “GLP1R agonist*” OR “GLP-1 analogue” OR “GLP1 analogue”)

AND NOT TITLE-ABS-KEY (“infant*” OR “child*” OR “gestational” OR “pregnanc*” OR “animal*” OR “rat” OR “rats” OR “mice” OR “mouse”)

Web of Science:

#1: (TS=("bariatric surgery" OR "bariatric intervention*" OR "obesity surgery" OR "bariatric procedure*" OR "gastric bypass" OR "biliopancreatic diversion" OR "RYGB" OR "sleeve gastrectomy" OR "BPD" OR "BPD-DS" OR "duodenal switch" OR "Single anastomosis duodeno-ileal bypass" OR "SADI-S" OR "SADI" OR "SIPS" OR "SASI" OR "gastric plication" OR "endosleeve" OR "endobarrier" OR "gastric clip*" OR "endoscopic bariatric therap*" OR "EBT" OR "endoscopic sleeve gastroplast*" OR "ESG" OR "intragastric balloon*" OR "endoluminal bypass liner*" OR "gastric band*")) NOT (TS=(“infant*” OR “child*” OR “gestational” OR “pregnanc*” OR “animal*” OR “rat” OR “rats” OR “mice” OR “mouse”)) AND LANGUAGE: (English) AND DOCUMENT TYPES: (Article)

Indexes=SCI-EXPANDED, SSCI, A&HCI, CPCI-S, CPCI-SSH, ESCI, CCR-EXPANDED, IC Timespan=All years

AND

#2: (TS=(“liraglutide” OR "semaglutide" OR "Tirzepatide" OR “glucagon-like peptide 1 receptor agonist*” OR “glucagon-like peptide-1 receptor agonist*” OR “GLP-1 receptor agonist*” OR “GLP1 receptor agonist*” OR “GLP-1R agonist*” OR “GLP1R agonist*” OR “GLP-1 analogue” OR “GLP1 analogue”)) AND LANGUAGE: (English) AND DOCUMENT TYPES: (Article)

Indexes=SCI-EXPANDED, SSCI, A&HCI, CPCI-S, CPCI-SSH, ESCI, CCR-EXPANDED, IC Timespan=All years

Final Search (#3): #1 AND #2
