# Supplementary material for: Use of incretin receptor agonists in patients submitted to metabolic bariatric surgery – a systematic review and meta-analysis
Source: Front Endocrinol (Lausanne). 2026 Aug 26;17:1870008. doi: 10.3389/fendo.2026.1870008 (PMC13553220; doi:10.3389/fendo.2026.1870008)
Supplement: Supplementary file 2 [file SupplementaryFile2.docx]

**Supplementary File 2** - Main results on the studies involving patients submitted to pharmacotherapy after metabolic bariatric surgery.

|  | Study Design | N | Type 2 Diabetes | Metabolic bariatric Surgery | Time since metabolic bariatric surgery | IWL or WR Definition | Dose after titration | Treatment duration | Comparison | Main outcomes |
| --- | --- | --- | --- | --- | --- | --- | --- | --- | --- | --- |
| Elhag & El Ansari, 2022 | Cohort | 145 | 20.7% | RYGB - 11.7% LSG - 85.5%  LAGB – 2.1%  SADI – 0.7% | 54.1 months | WR: >10kg from nadir  IWL: <50% of excess weight lost at 18 months after intervention | Liraglutide 3.0 mg daily | 12 months | Primary vs revisional;  Start vs 6 months vs 12 months | Primary Surgery: TWL 5.97% and 6.93% (6 and 12 months, respectively). At 12 months, 60% of patients lost at least 5% of total body weight.  Revision Surgery: TWL 6.41% and 4.99% (6 and 12 months, respectively). At 12 months, 48% of patients lost at least 5% of total body weight. |
| Muratori et al., 2022 | Case Series | 62 | ND | LSG - 37% LAGB - 35.6% RYGB - 27.4% | 70.7 ± 43.7 months | WR: 10-15% from minimum weight  IWL: if unsatisfied with WL | Liraglutide 3.0 mg daily | 10.5 ± 4.4 months | Before vs after liraglutide;  LGB vs LSG vs RYGB | Average BMI loss of 5.1kg/m^2^. No differences in regard to the type of surgery. |
| Horber & Steffen, 2021 | Cohort | 34 | 5.8% | RYGB - 100% | 6 -13 (9 ± 5) years | WR: >10% from nadir | Liraglutide 3.0 mg daily | 24 months | - | Control group: average BMI change of +0.1kg/m^2^.  Liraglutide group: average BMI change of -4.8kg/m^2^. |
| Rubio & Ramos-Leví, 2021 | Cohort | 23 | ND | ND | 72 months | WR: >15% from nadir | Liraglutide 3.0 mg daily or every other day | 12 months | Daily vs alternate-day | %TWL - 17.6% ± 5.5  >85% of patients lost regained weight. Every other day dosing after 6 months of daily administration was as effective as daily dosing. |
| Miras et al., 2019 | RCT | 48 | 100% | RYGB - 79.2% VSG - 20.8% | 3.8 ± 2.0 years | ND | Liraglutide 1.8 mg daily | 26 weeks | Liraglutide vs placebo | **Primary outcome**: change in HbA1c.  **Secondary outcome**: changes from baseline in bodyweight, systolic and diastolic blood pressure, among other parameters. Average weight change difference from baseline to week 26 for liraglutide vs placebo of -2.34 kg. |
| Wharton et al., 2019 | Cohort | 117 | ND | RYGB - 45.3% Gastric banding - 42.7% Gastric sleeve - 12% | 8 years | WR: + 10-25% of excess weight or total weight lost | Liraglutide 3.0 mg daily | 7.6 ± 7.1 months | RYGB vs Gastric banding vs Gastric sleeve | Average TWL - 5.5% ± 6.2 (6.3kg). No differences in regard to the type of surgery. WL after 12 months of treatment greater than at 6 months. |
| Rye et al., 2018 | Cohort | 20 | 25% | RYGB - 35% LSG - 35% VBG - 15% LAGB - 15% | 76.3 ± 72.9 months | WR: >10% from nadir  IWL: <20% WL from baseline | Liraglutide 2.9 mg mean daily dose | 28 weeks | - | **Primary outcome**: %TWL and median BMI change 9.7% and -4.7kg/m2, respectively 50% of patients attained a body weight lower than nadir. |
| Gorgojo-Martínez et al., 2016 | Cohort | 15 | 100% | RYGB - 53.3% BPD - 20% VBG - 13.3%  SG - 6.7%  LAGB - 6.7% | 5.2 years | WR: >5% from nadir  IWL: BMI >35 kg/m^2^ or %EWL <50% | Liraglutide1.2 mg daily and 1.8 mg daily if insufficient glycemic response or weight loss (91.7%) | 24 months | Previous BS vs no BS | Average WL of 3.4kg.  BMI change of -1.2kg/m^2^. |
| Pajecki et al., 2013 | Case Series | 15 | ND | RYGB - 60% LAGB - 26.6% SG - 6.7% BPD - 6.7% | 5.3 years | WR: >15% from nadir  IWL: <50% of excess weight | Liraglutide 1.2 to 1.8 mg daily, with this last one in 53.3% of the participants | 12.5 ± 4.7 weeks | Before vs after liraglutide | %TWL of 7.38%. |
| Lofton et al., 2025 | RCT | 89 | ND | RYGB – 100% | 18-120 months | WR: >10% from nadir | Liraglutide 3.0 mg daily | 56 weeks | Liraglutide vs placebo | **Co-primary outcomes**: proportions of subjects losing ≤5%, ≤10%, ≤20%, and ≥20% of baseline BW after 52 weeks and proportion who were able to achieve their postoperative NW.  Average %TBWL change was -8.8 and 32.8% EBWL. 17% achieved ≥5% TBWL, 51% achieved ≥10% TBWL and 26% achieved ≥15% TBWL.  **Secondary outcomes**: changes from baseline to 12 months. |
| Jamal et al., 2024 | Cohort | 57 | 3.51% | SG – 100% | ± 10 years | WR: ≥10% from the lowest post-SG weight  IWL: <20% WL from preoperative weight | Liraglutide 3.0 mg daily | ± 3 months | Before vs after treatment | Average WL of 5.49 ± 6.31, corresponding to a loss of 6.20% of pre-treatment weight. Patients who tolerated ≥2.4 mg of liraglutide recorded a higher mean WL of 8.10%. |
| Vinciguerra et al., 2023 | Cohort | 59 | 0% | SG – 64%  One-anastomosis gastric bypass – 36% | 20 ± 6 months | WR: gain of at least 15% of the WL after metabolic bariatric surgery  IWL: initial WL <50% of EWL | Liraglutide mean dose 2.4 mg daily | 24 weeks | Before vs after liraglutide;  Male vs female, SG vs OAGB, IWL vs WR | Average WL of 8.4% ± 3.6. 11.8% of the patients lost between 0 and 5%, while 59.3% lost between 5 and 10%, 20.3% lost between 10 and 15%, and 8.5% lost more than 15% of their body weight. |
| Suliman et al., 2019 | Cohort | 189 | ND | SG – 63%  RYGB – 25%  Other procedures – 12% | ± 4 years | ND | Liraglutide 3.0 mg daily | ≥16 or ≥28 weeks | SCALE vs ≥16 vs ≥28 weeks | Average WL was 6.1% of the patients treated for ≥16 weeks; Average WL was 5.6% for the RYGB group and 3.3% for the SG group of the patients treated for ≥28 weeks. |
| Mok et al., 2023 | RCT | 31 | 14% | RYGB – 6%  SG – 94% | At least 1 year | IWL: 20% or less total body WL from the day of surgery | Liraglutide 3.0 mg daily | 24 weeks | Liraglutide vs placebo | Estimated change in percentage body weight from baseline to week 24 was −8.82 (4.94) with liraglutide. 71.9% of the patients lost 5% or more of their body weight. |
| Colbourne et al., 2023 | Cohort | 68 | 5.9% | LSG – 44.1%  Gastric band - 26.5%  RYG bypass -22.1%  Others – 7.4%  Revisional – 29.4% | At least 1 year after LSG and RYGB and at least 2 years after LGB | IWL: BMI> 35 kg/m^2^ (all patients), >25% TWL (LSG; RYGBP), >20% TWL (LAGB) | Liraglutide 1.8 – 3.0 mg daily | 3 months | - | TBWL of 5.30% (2.23–8.35%). 22.1% of the patients showed good response define as ≥10% TBWL. Patients with a revisional surgery presented an average of 6.5% of TBWL. |
| Firkins et al., 2024 | Cohort | Semaglutide – 310 Liraglutide - 1720 | 38.86% (Semaglutide – 87.10% and Liraglutide 86.05%) | SG – 69.4%  RYGB – 30.6% | Within 5 years | ND | ND | ND | AOMs | OR (95%, CI)  Semaglutide – individuals with >30kg/m^2^ 2.72 (2.17-3.4) and >40kg/m^2^ 2.23 (1.73-2,87)  Liraglutide – individuals with >30kg/m^2^ 1.93 (1.75-2.13), >40kg/m^2^ 1.97 (1.78-2.19). |
| Stanford et al., 2018 | Cohort | Liraglutide - 7 | 51% | SG – 31.4%  RYGB – 68.6% | SG – 26.2 months  RYGB – 38.5 months | ND | ND | ND | SG vs RYGB | Only medication to show a significant negative beta coefficient in the linear regression analysis using medication as a predicator of WL (β=-16.07, p=0.009), and CI=-27.76 to -4.38). |
| Jamal et al., 2024 | Cohort | Semaglutide – 70  Tirzepatide - 45 | Semaglutide – 1.4%  Tirzepatide - 2.2% | SG – 100% | 71.8 ± 51.1 months | WR: a priori ≥ 10% weight recurrence from the nadir post-SG weight | 68.6% with ≥1.0 mg of semaglutide and 63.8% with ≥10.0 mg of tirzepatide | ND | Tirzepatide vs semaglutide | Semaglutide – Average WL of 6.0 ± 3.6 % at 3 months and 10.3 ± 5.9 % at 6 months.  Tirzepatide – Average WL of 9.3 ± 4.3 % at 3 months and 15.5 ± 6.3 % at 6 months. |
| Bahdi et al., 2025 | Cohort | 68 (59 Semaglutide, 7 Tirzepatide) | 44.1% | SG – 100% | 52.4 ± 33 .8 months | Weight recidivism: ≥25% weight gain from nadir weight following SG | Semaglutide – 1.25 mg daily  Tirzepatide – 7.5 mg daily | ± 9.3 months | Prior SG+GLP1/GIP RA vs Prior SG+R-ESG vs Intact stomach + GLP1/GIP RA | **Primary outcomes**: TBWL, EWL and adverse events  Tirzepatide TWBL – 3.6% ± 4.2 (3m), 13.5% ± 5.6 (6m), 13.2% ± 5 (12m)  Semaglutide TWBL – 4.1% ± 4.1 (3m), 6.3% ± 5.5 (6m), 8.1% ± 7 (12m)  **Secondary outcomes**: change in metabolic parameters |
| Jensen et al., 2023 | Cohort | Liraglutide – 29  Semaglutide - 21 | 18% | RYGB – 82%  SG – 10%  Distal RYGB – 8% | 72 months | WR: any weight gain following the weight nadir at least 12 months after BS | Liraglutide -3.0 mg daily  Semaglutide - 1.0 mg weekly injection or 14.0 mg daily oral intake | 6 months | Liraglutide vs semaglutide | **Primary outcomes**: change in total body weight and BMI and percentage of weight regain lost  TBWL of 8.8% at 6 months, with 7.3% (3.1, 10.3) for liraglutide and 9.8% (8.2, 13.0) for semaglutide. A TBWL of ≥5% of baseline weight was achieved by 69%, ≥10% by 31%, and ≥15% by 3.5% of the patients with liraglutide. A TBWL of ≥5% was achieved by 85.7%, ≥10% by 47.6% and ≥15% by 23.8% of the patients with semaglutide  **Secondary outcomes**: safety based on therapy-related adverse events |
| Jensen et al., 2025 | Cohort | Liraglutide – 22  Semaglutide - 18 | 25% | RYGB – 77.5%  SG – 7.5%  Distal RYGB – 5% | 74.5 months | WR: any weight gain after having reached the weight nadir, at least 15 months after BS | Liraglutide - 3.0 mg daily  Semaglutide – 1.0 mg weekly | 12 months | Pre-surgery vs post-surgery weight nadir vs GLP1-RA initiation vs 6 months treatment vs 12 months treatment | **Primary outcomes**: changes in total body weight and BMI, and proportion of body weight regain and excess body weight lost.  TBWL of 10.5 corresponding to 41.7% of their excess weight. Significant difference in WL between liraglutide and semaglutide (-3.1 (-4.7, -2.0) kg/m^2^ vs -4.7 (-6.0, -3.7) kg/m^2^).  **Secondary outcomes**: GLP1-RA-related adverse events and prevalence of obesity-related comorbidities. |
| Murvelashvili et al., 2023 | Cohort | 207 (115 semaglutide, 92 liraglutide) | 7.73% | SG – 50%  RYGB – 29%  AGB – 21% | Liraglutide - 8.24 ± 7.10 years  Semaglutide – 7.81 ± 6.73 years | ND | Liraglutide – 3.0 mg daily  Semaglutide – 1.0 mg weekly | 3 months | Liraglutide vs semaglutide | Average weight changes at 12 months of -12.92 in the semaglutide group and -8.77% in the liraglutide group. At 12 months, 77.4% of patients in the semaglutide group achieved ≥5% WL compared to 67.4% in the liraglutide group.  People achieving ≥10%, ≥15% and ≥20% WL was 50.4%, 27.8% and 12.2% for the semaglutide group and 32.6%, 15.2%, and 5.4% for the liraglutide group. |
| Lautenbach et al., 2022 | Cohort | 44 | 0% | SG -65.9%  RYGB – 34.1% | 64.7 ± 47.6 months | WR: continuous WR after an initial successful WL (defined as EWL>50%)  IWL: achieving a nadir weight with EWL<50% after surgery | Semaglutide 0.5 mg weekly | 6 months | Baseline vs 3 M-FU vs 6-M FU | TWL -6.0 ± 4.3 % at 3 months and -10.3 ± 5.5 % at 6 months. At 3 months WL was >5% in 61%, >10% in 16% and >15% in 2% of patients. At 6 months WL was >5% in 85%, >10% in 45% and >15% in 5% of patients. |
| Lautenbach et al., 2023 | Cohort | 29 | 0% | RYGB – 37.9%  SG – 62.1% | 64.5 ± 51.9 months | WR: continuous WR after an initial successful WL (defined as EWL>50%)  IWL: achieving a nadir weight with EWL<50% after surgery | Semaglutide 1.0 mg weekly | 12 months | Baseline vs 6M-FU vs 9M-FU vs 12M-FU;  RYGB vs SG | **Primary outcomes**: WL  Average TWL added up to 14.7% ± 8.9% at 12 months and 10.3% ± 6.4% at 6 months. 89.7% of patients reached >5% WL, 62.1% reached >10%, 34.5% reached >15%, 24.1% reached >20% and 17.2% reached >25%.  **Secondary outcomes**: change in BMI, HbA1c, lipid profile, high sensitive C-reactive protein and liver enzymes. |
| Bonnet et al., 2024 | Cohort | 36 | 23% | SG – 71.8%  RYGB – 33.3% | 8.4 years | WR  IWL: EWL<50% 18 months post-BS | Semaglutide 2.4 mg weekly | 24 weeks | BS + group vs BS - group | 9.8% ± 5.7% WL and - 23.2% ± 14.9% EWL.  72% of patients lost at least 5% of their initial BW, 36% lost at least 10% and 8% lost at least 15%. |
| Kanai et al., 2024 | Cohort | 29 | 100% | LSG – 100% | 1 year | Weigh recurrence: 5% weight recurrence from nadir | Semaglutide 1.0 mg weekly | 12 months | Before LSG vs 12 months after LSG vs before semaglutide vs after 12 months semaglutide | **Primary outcomes**: change in HbA1c and BMI.  At 12 months average BW of 100 kg. Mean body weight decreased from 104.6 to 100.0 kg and mean BMI decreased from 37.7 to 36.1 kg/m^2^.  **Secondary outcomes**: change in skeletal muscle ratio and various measured nutritional metrics. |
| Stoll et al., 2025 | Cohort | 21 | 0% | RYGB – 48%  SG – 52% | 7.2 ± 4.3 years | WR: continuous weight increase after an initial successful WL (EWL>50% 18 months post-MBS)  IWL: achieving a nadir weight with EWL <50% 18 months after surgery | Tirzepatide 5.0 mg weekly | 6 months | Baseline vs 1M-FU vs 3M-FU vs 6M-FU | At 6 months, TWL added up to 12 ± 3.4% and all patients reached ≥5% WL, 76.5% reached ≥10% WL, and 23.5% reached ≥15% WL. |

BMI, body mass index; BPD, biliopancreatic diversion; EWL, excess weight loss; HbA1c, hemoglobin A1c; IWL, insufficient weight loss; LGB, laparoscopic gastric banding; LSG, laparoscopic sleeve gastrectomy; ND, not determined; NW, nadir weight; OAGB, one anastomosis gastric bypass; RCT, randomized controlled trial; RYGB, roux-en-y gastric bypass; SADI, single anastomosis duodenal switch; SG, sleeve gastrectomy; TWL, total weight loss; VBG, vertical banded gastroplasty; VSG, vertical sleeve gastrectomy; WL; weight loss; WR, weight regain.
