# Supplementary material for: Use of incretin receptor agonists in patients submitted to metabolic bariatric surgery – a systematic review and meta-analysis
Source: Front Endocrinol (Lausanne). 2026 Aug 26;17:1870008. doi: 10.3389/fendo.2026.1870008 (PMC13553220; doi:10.3389/fendo.2026.1870008)
Supplement: Supplementary file 3 [file SupplementaryFile3.docx]

**Supplementary File 3 –** Quality assessment

Summary of the Jahad Scale scores of the randomized controlled trials included in the systematic review.

| **Jahad Scale for randomized controlled trials** | | | | |
| --- | --- | --- | --- | --- |
| **First author and year** | **Randomization** | **Blinding** | **An account of all patients** | **Total** |
| Miras A.D., 2019 | 2 | 2 | 1 | 5 |
| Lofton H.F., 2025 | 2 | 2 | 1 | 5 |
| Mok J., 2023 | 2 | 2 | 1 | 5 |

Summary of the Methodological index for non-randomized studies (MINORS) scores of the Cohort Studies included in the systematic review.

| **Methodological index for non-randomized studies (MINORS) for Cohort Studies** | | | | | | | | | | | | | |
| --- | --- | --- | --- | --- | --- | --- | --- | --- | --- | --- | --- | --- | --- |
|  |  |  |  |  |  |  |  |  | **Additional criteria in the case of comparative study** | | | |  |
| **First author and year** | **1** | **2** | **3** | **4** | **5** | **6** | **7** | **8** | **9** | **10** | **11** | **12** | **Total score** |
| Elhag W., 2022 | 2 | 2 | 0 | 2 | 1 | 2 | 1 | 0 |  |  |  |  | **10/16** |
| Horber F.F., 2021 | 2 | 2 | 2 | 2 | 0 | 2 | 2 | 0 |  |  |  |  | **12/16** |
| Rubio M.A., 2021 | 2 | 2 | 2 | 2 | 0 | 2 | 1 | 0 |  |  |  |  | **11/16** |
| Wharton S., 2019 | 2 | 2 | 0 | 2 | 0 | 2 | 1 | 0 |  |  |  |  | **9/16** |
| Rye P., 2018 | 2 | 2 | 0 | 2 | 0 | 2 | 1 | 0 |  |  |  |  | **9/16** |
| Gorgojo-Martínez J.J., 2016 | 2 | 1 | 0 | 2 | 0 | 2 | 1 | 0 | 2 | 2 | 1 | 2 | **15/24** |
| Jamal M., 2024 | 2 | 1 | 0 | 2 | 0 | 1 | 1 | 0 |  |  |  |  | **7/16** |
| Vinciguerra F., 2023 | 2 | 1 | 0 | 2 | 0 | 2 | 2 | 2 |  |  |  |  | **11/16** |
| Suliman M., 2019 | 2 | 2 | 2 | 2 | 0 | 1 | 1 | 0 |  |  |  |  | **10/16** |
| Colbourne J.R.M., 2023 | 2 | 2 | 1 | 2 | 0 | 2 | 2 | 0 |  |  |  |  | **11/16** |
| Stanford F.C., 2018 | 2 | 1 | 0 | 2 | 0 | 2 | 1 | 0 |  |  |  |  | **8/16** |
| Jamal M., 2024 | 2 | 2 | 1 | 2 | 0 | 1 | 1 | 0 |  |  |  |  | **9/16** |
| Bahdi F., 2025 | 2 | 2 | 0 | 2 | 0 | 2 | 1 | 0 |  |  |  |  | **9/16** |
| Jensen A.B., 2023 | 2 | 1 | 0 | 2 | 0 | 2 | 1 | 0 |  |  |  |  | **8/16** |
| Jensen A.B., 2025 | 2 | 1 | 0 | 2 | 0 | 2 | 1 | 0 |  |  |  |  | **8/16** |
| Murvelashvili N., 2023 | 2 | 1 | 0 | 2 | 0 | 2 | 1 | 0 |  |  |  |  | **8/16** |
| Firkins S.A., 2024 | 2 | 2 | 0 | 2 | 0 | 2 | 1 | 0 |  |  |  |  | **9/16** |
| Lautenbach A., 2022 | 2 | 1 | 0 | 2 | 0 | 1 | 1 | 0 |  |  |  |  | **7/16** |
| Lautenbach A., 2023 | 2 | 1 | 0 | 2 | 0 | 2 | 1 | 0 |  |  |  |  | **8/16** |
| Bonnet J., 2023 | 2 | 1 | 0 | 2 | 0 | 1 | 2 | 0 |  |  |  |  | **8/16** |
| Kanai R., 2024 | 2 | 1 | 0 | 2 | 0 | 2 | 2 | 0 |  |  |  |  | **9/16** |
| Stoll F., 2025 | 2 | 1 | 1 | 2 | 0 | 2 | 1 | 0 |  |  |  |  | **9/16** |

1. A clearly stated aim

2. Inclusion of consecutive patients

3. Prospective collection of data

4. Endpoints appropriate to the aim of the study

5. Unbiased assessment of the study endpoint

6. Follow-up period appropriate to the aim of the study

7. Loss to follow up less than 5%

8. Prospective calculation of the study size

Additional criteria in the case of comparative study

9. An adequate control group

10. Contemporary groups

11. Baseline equivalence of groups

12. Adequate statistical analyses

Summary of the Joanna Briggs Institute Critical Appraisal Checklist scores of the Case Series included in the systematic review.

| **Joanna Briggs Institute Critical Appraisal Checklist for Case Series** | | | | | | | | | | | |
| --- | --- | --- | --- | --- | --- | --- | --- | --- | --- | --- | --- |
| **First author and year** | **1** | **2** | **3** | **4** | **5** | **6** | **7** | **8** | **9** | **10** | **Total** |
| Muratori F., 2022 | x | x | x |  |  | x | x | x |  | x | 7 |
| Pajecki D., 2013 |  | x | x |  |  | x | x | x |  | x | 6 |

1. Were there clear criteria for inclusion in the case series?

2. Was the condition measured in a standard, reliable way for all participants included in the case series?

3. Were valid methods used for identification of the condition for all participants included in the case series?

4. Did the case series have consecutive inclusion of participants?

5. Did the case series have complete inclusion of participants?

6. Was there clear reporting of the demographics of the participants in the study?

7. Was there clear reporting of clinical information of the participants?

8. Were the outcomes or follow up results of cases clearly reported?

9. Was there clear reporting of the presenting site(s)/clinic(s) demographic information?

10. Was statistical analysis appropriate?
